# Author Correction: Molecular engineering of safe and efficacious oral basal insulin

**DOI:** 10.1038/s41467-020-18106-3

**Published:** 2020-08-20

**Authors:** Frantisek Hubálek, Hanne H. F. Refsgaard, Sanne Gram-Nielsen, Peter Madsen, Erica Nishimura, Martin Münzel, Christian Lehn Brand, Carsten Enggaard Stidsen, Christian Hove Claussen, Erik Max Wulff, Lone Pridal, Ulla Ribel, Jonas Kildegaard, Trine Porsgaard, Eva Johansson, Dorte Bjerre Steensgaard, Lars Hovgaard, Tine Glendorf, Bo Falck Hansen, Maja Kirkegaard Jensen, Peter Kresten Nielsen, Svend Ludvigsen, Susanne Rugh, Patrick W. Garibay, Mary Courtney Moore, Alan D. Cherrington, Thomas Kjeldsen

**Affiliations:** 1 Novo Nordisk A/S, Novo Nordisk Park 1, 2760 Maaloev, Denmark; 2 Vanderbilt University, Nashville, TN USA

**Keywords:** Drug discovery, Endocrine system and metabolic diseases, Molecular medicine

Correction to: *Nature Communications* 10.1038/s41467-020-17487-9, published online 27 July 2020.

The original version of this Article omitted the following from the Acknowledgements:

“We are grateful to Janos Tibor Kodra and Jette Lenstrup, both of Novo Nordisk A/S, who conceived and provided the OI216 insulin analogue. Furthermore, we are appreciative of Inger Lautrup-Larsen, Novo Nordisk A/S, contributions with recombinant expressing, as well as many colleagues’ producing, purifying and quantifying the compounds used in this study”.

This has now been corrected in both the PDF and HTML versions of the Article.

