# Supplementary material for: Molecular engineering of safe and efficacious oral basal insulin
Source: Nat Commun. 2020 Jul 27;11:3746. doi: 10.1038/s41467-020-17487-9 (PMC7385171; doi:10.1038/s41467-020-17487-9)
Supplement: Supplementary file 1 — Supplementary Information [file 41467_2020_17487_MOESM1_ESM.pdf]

Molecular Engineering of Safe and Efficacious Oral Basal Insulin

Hubálek *et al*

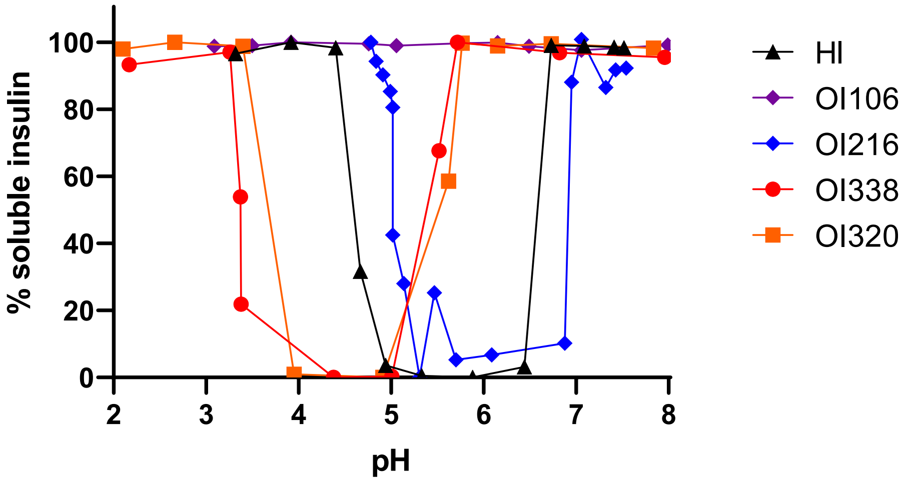

Supplementary Figure 1 Solubility of insulin analogues in aqueous solution at different pH. The pH of insulin aqueous solution (~600 μM) was adjusted using dilute HCl or NaOH solutions in the range from 2-8. Samples were equilibrated for 24 h at ambient temperature. Insulin concentration (absorbance at 276 nm) and pH were measured in supernatants after centrifugation (20 min, 20000 g, 20°C).

Supplementary Figure 2. X-ray structures of OI338 and OI320. Zinc free crystal structures of OI338 and OI320 were determined to 1.5 Å resolution and the fold of the insulin monomer was found to be conserved as compared to human insulin[1] (Supplementary information Fig. 2a and b). Structural alignment of Cα atoms in Pymol[2] using default parameters results in root mean square deviations of 0.37Å for OI338 (44 atoms) and 0.56Å for OI320 (41 atoms), respectively, when aligned to zinc free human insulin (Protein Data Bank ID 6S34). The larger value for OI320 is expected since this structure is determined in a different crystal form than zinc free human insulin.

OI338 crystallizes in the same crystal form as zinc free human insulin, which has an insulin monomer in the asymmetric unit and where the crystal symmetry may be applied to assemble the insulin dimer (Supplementary information Fig. 2a). The modified residues (A14Glu and B25His) show nice difference in the electron density while the fatty acid moiety covalently attached to B29 Lys is disordered.

OI320 also has an insulin monomer in the asymmetric unit. However, in this crystal form, the insulin dimer is not formed, but instead one can find a trimeric assembly where the fatty acid moieties from three insulin molecules are placed at the centre aligned with the insulin dimerization surface (Supplementary information Fig. 2 c). This is likely a result of the desB27 modification which weakens the dimerization by removing one of the residues at both ends of the antiparallel beta strand which normally is formed upon insulin dimerization (Supplementary information Fig. 2 a). The modified residues are nicely defined in the crystal structure and the only disordered part is the polyethylene glycol like linker of the side chain.

a b c

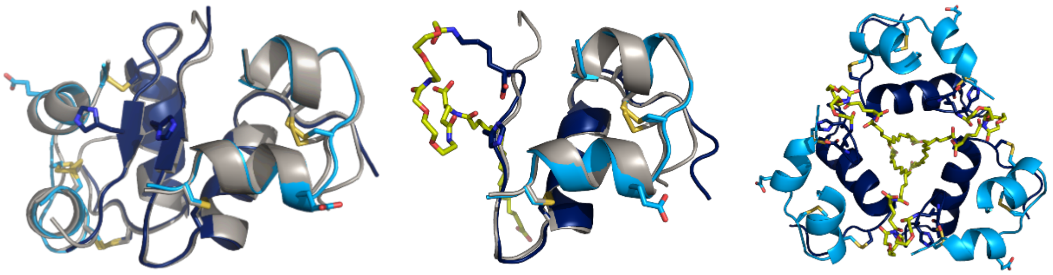

Supplementary Figure 2 Cartoons of (a) OI338 dimer and (b) OI320 monomer superimposed to zinc free human insulin (grey, Protein Data Bank ID 6S34). OI338 and OI320 are displayed with the A chain in light blue and the B chain in dark blue. OI320 packs in a trimeric arrangement (c) in the crystal, where the fatty acid side chains (yellow) from three insulin monomers come together and thereby shields the hydrophobic patches on insulin which usually are part of dimerization in addition to protecting the fatty acid from exposure to the solvent. The side chains of the A14Glu and B25His substitutions of OI338 and OI320 are displayed in stick representations.

	OI 338	OI 320
**Data collection**		
Space group	$I2_13$	P321
Cell dimensions		
a, *b*, *c* (Å)	77.48 77.48 77.48	44.99 44.99 45.88
α, β, γ (°)	90 90 90	90 90 120
Resolution (Å)	31.63 - 1.511 (1.565 - 1.511)*	45.88 - 1.5 (1.554 - 1.5)
R_{merge}	0.05816 (0.5101)	0.05618 (1.635)
$I / \sigma I$	54.75 (4.25)	21.02 (1.26)
Completeness (%)	99.70 (97.40)	99.85 (98.85)
Redundancy	25.2 (14.9)	9.3 (7.8)
**Refinement**		
Resolution (Å)	31.63 - 1.511	45.88 - 1.5
No. reflections	12255	8934
R_{work} / R_{free}	0.1799/0.2122	0.1873/0.2100
No. atoms	458	497
Protein	418	406
Ligand/ion	8	55
Water	32	36
B-factors	40.03	33.65
Protein	39.17	21.20
Ligand/ion	51.22	48.00
Water	48.51	39.47
R.m.s. deviations		
Bond lengths (Å)	0.009	0.013
Bond angles (°)	1.07	1.23

Supplementary Table 1: Data collection and refinement statistics

*Values in parentheses are for highest-resolution shell.

Supplementary Figure 3. Insulin receptor affinities. Solubilized receptors. Enrichment of solubilized insulin and insulin-like growth factor 1 (IGF1) receptors expressed in Baby Hamster Kidney (BHK) cells by wheat germ agglutinin agarose chromatography and subsequent radio-ligand binding by scintillation proximity assays (SPA)[3-6]. Using solubilized human insulin receptors, the affinities of insulin analogues for the A-isoform of the human insulin receptor were determined in a competition SPA assay in the absence or presence of 1.5% HSA. All concentration-response relations from binding assays were analysed by application of a four-parameter logistic model[7, 8] assuming common slope, basal and maximal response level of the curves for human insulin and the insulin analogue. Affinities for insulin analogues were reported relative to human insulin (equation 1) [EC_{50}(human insulin)/EC_{50}(Analog)×100%] within each plate. A typical example of a competition assay is shown in Supplementary data Fig. 3.

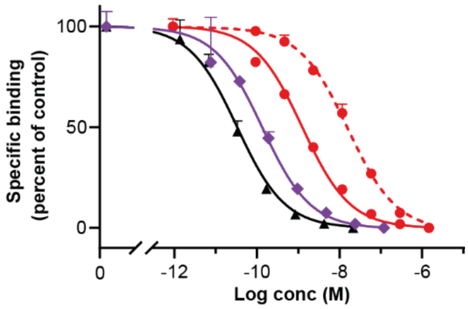

Supplementary data Fig. 3. Displacement of ^{125}I-human insulin from wheat germ agglutinin enriched, solubilized human insulin receptors by human insulin (▲black), OI106 (♦purple), OI338 (• red), OI338 with 1.5% HSA (• red dashed). Data are from a representative experiment (mean, SD, n=4).

Supplementary Table 2. Summary of receptor affinities. Solubilized receptors. Preparation of solubilized insulin and insulin-like growth factor 1 (IGF1) receptors and subsequent radio-ligand binding by scintillation proximity assays (SPA) was accomplished as described for Supplementary data Fig. 3. Membrane-associated receptors. Isolation of plasma membranes from BHK cells expressing human insulin receptors and subsequent radio-ligand binding to membrane-associated receptors by SPA[6].

Receptor affinities of insulin analogues relative to human insulin are shown in Supplementary Table 2. Using solubilized human insulin receptors, the affinities of insulin analogues for the A-isoform of the human insulin receptor were determined in a competition SPA assay in the absence or presence of 1.5% HSA. The affinities of insulin analogues were also determined using solubilized insulin-like growth factor 1 (IGF1) receptors in the absence of HSA and membrane preparations of BHK cells expressing cloned recombinant insulin receptor isoforms of human origin in the presence of 0.1% HSA. Affinities for human IGF1 receptors were generally lower, yet proportional to affinities for human insulin receptors. Moreover, human insulin and insulin analogues display identical affinities for the short (A) and long (B) isoform of human insulin receptors (hIR-A and hIR-B, respectively).

Receptor	hIR-A sol			hIR-A sol			hIGF1R sol			hIR-A mem			hIR-B mem		
HSA (%)	0			1.5			0			0.1			0.1		
**Analog**	Mean	95% CI	n	Mean	95% CI	n	Mean	95% CI	n	Mean	95% CI	n	Mean	95% CI	n
OI216	14	11 to 16	13	0.76	0.62 to 0.91	12	4.4	-	2	1.8	1.5 to 2.1	5	1.5	1.1 to 2.2	3
OI338	2.4	2.2 to 2.6	24	0.10	0.095 to 0.11	18	0.73	0.66 to 0.80	12	0.16	0.13 to 0.20	39	0.18	-	2
OI106	24	21 to 27	10	27	21 to 35	5	N.D.	-	-	27	22 to 33	12	28	27 to 29	5
OI189	30	19 to 47	4	36	33 to 39	6	N.D.	-	-	47.	39 to 55	3	50.	41 to 63	3
OI320	3.2	2.8 to 3.6	13	0.26	0.22 to 0.31	12	1.4	1.1 to 1.9	6	0.62	0.35 to 1.1	6	0.64	0.35 to 1.2	6

Supplementary Table 2. Summary of receptor affinities (percent relative to human insulin)

Affinities of insulin analogues relative to human insulin for solubilized (indicated by sol) and membrane-associated insulin and IGF1 receptor (indicated by mem) affinities. N.D. not determined.

Supplementary Figure 4. Metabolic responses. Glycogen synthesis in primary hepatocytes. Hepatocytes were isolated from healthy male Sprague-Dawley rats (8 weeks old, 200–300 g body weight) using a standard collagenase-perfusion protocol[9]. After 24 h of insulin/insulin analogue stimulation, cellular glycogen was digested with amylo-glucosidase (Sigma) for 3 h at 37°C and glucose measured using a hexokinase/Glu6PDH assay[10].

A typical example of a dose-response curve for stimulation of glycogen synthesis is shown in Supplementary data Fig. 4 and the calculated potencies are listed in Supplementary data Table 3. The maximal effect of insulin analogues was the same as for human insulin indicating that the insulin analogues are full insulin receptor agonists. Additionally, the effect of HSA was examined by measuring the relative potency with and without the addition of 1% HSA. The dose-response curves for the analogues were greatly right-shifted in the presence of added HSA.

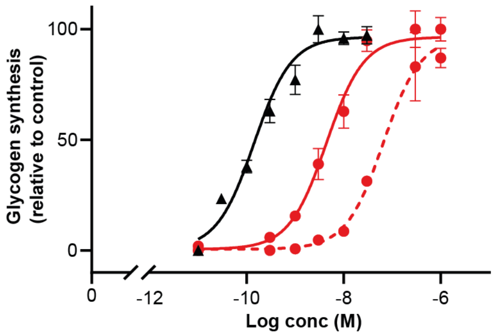

Supplementary data Fig. 4**.** Stimulation of glycogen synthesis in primary rat hepatocytes for human insulin (▲ black), OI338 (• red), and OI338 with 1% HSA (• red dashed). Data are from a representative experiment (mean, SD, n=3).

Supplementary Table 3. Summary of metabolic responses and signal transduction.

Lipogenesis: Stimulation of lipogenesis in primary rat adipocytes was determined using the free fat cell assay[11, 12].

Glycogen synthesis: Glycogen synthesis in primary rat hepatocytes was measured as described in Supplementary data Fig. 4.

Phosphorylation of IR: Insulin-induced stimulation of IRTyr1158 phosphorylation in Chinese Hamster Ovary cells over-expressing the human insulin receptor (CHO-hIR cells) was determined by ELISA[13].

Potencies relative to human insulin were analysed using a four-parameter logistic model [7, 8] assuming common slope, basal and maximal response level of the curves for human insulin and the insulin analogues.

The data show that the insulin analogues give the same metabolic response relative to human insulin in both primary adipocytes and hepatocytes at 1% HSA. The effect of HSA was examined by measuring the relative potency at different HSA concentrations. As expected, the results show a clear dependency on the HSA concentration in both primary adipocytes and hepatocytes.

Analog	Lipogenesis (0.1% HSA)			Lipogenesis (1% HSA)			Glycogen hepatocytes (0% HSA)			Glycogen hepatocytes (1% HSA)			pIR-Tyr1158 CHO (0.1% HSA)		
	Mean	95% CI	n	Mean	95% CI	N	Mean	95% CI	n	Mean	95% CI	n	Mean	95% CI	n
OI338	0.41	0.37 to 0.45	3	0.10	0.10 to 0.11	3	4.4	3.6 to 5.4	7	0.13	0.12 to 0.16	7	0.39	0.26 to 0.57	3
OI106	-	-	-	29	28 to 30	10	-	-	-	-	-	-	24	17 to 35	3
OI320	0.37	0.33 to 0.43	6	0.19	0.17 to 0.22	5	4.7	4.2 to 5.3	6	0.19	0.17 to 0.22	6	1.6	1.2 to 2.3	3

Supplementary Table 3; Summary of metabolic responses and signal transduction of insulin analogues and human insulin. Potencies are listed as percent relative to human insulin.

Supplementary Figure 5. Albumin binding. Binding of [^{125}I]-Tyr-labelled, acylated insulin analogues via the fatty acid group at ε-amino B29Lys to agarose-immobilized serum albumin was performed as previously reported for [^{125}I]-TyrA14-labelled insulin detemir[14]. The albumin binding of OI338 was examined by monitoring the binding of ^{125}I-OI338 to human serum albumin in an equilibrium binding assay. The affinity of OI338 for albumin was estimated to be 4.4 ± 0.2-fold relative to insulin detemir (Supplementary data Fig. 5).

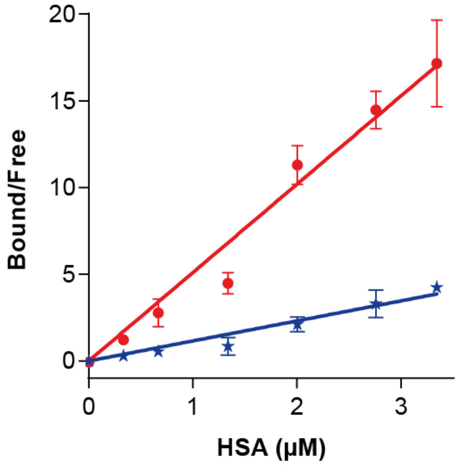

Supplementary Figure 5. Binding isotherms from equilibrium-binding of ^{125}I-OI338 (• red) and 125I-insulin detemir (* blue) to agarose-immobilized HSA. Data are presented as mean +/- SD from n=4 independent experiments.

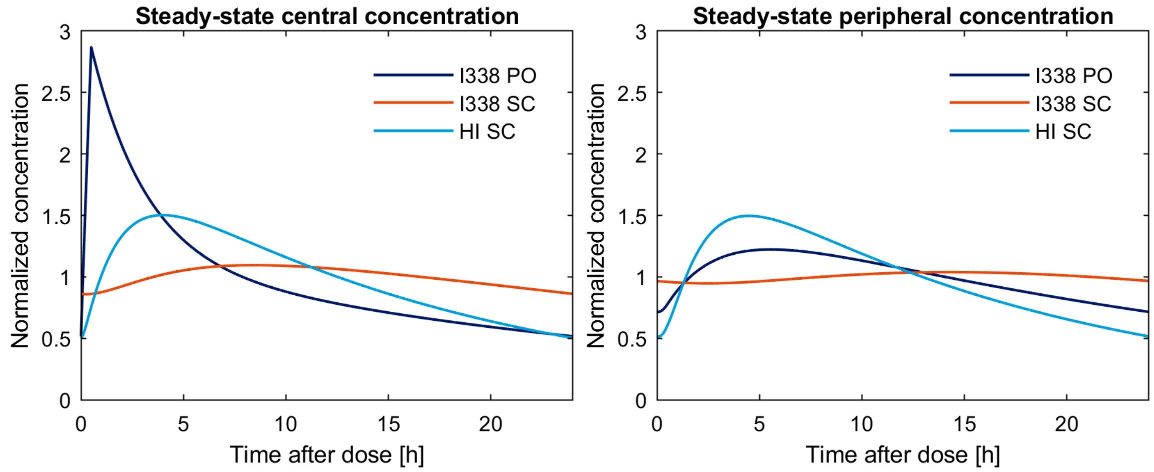

Supplementary Figure 6. The figures show simulations of *SC* and *PO* administration with either human insulin or OI338 in the central (plasma) and peripheral (tissue) compartments, respectively. The simulations are normalized to the AUC for each plot and are based on data on the insulin formulations tested in dogs. The plots show that *PO* OI338 have an initial phase with a high central-to-peripheral ratio of insulin which *SC* OI338 and *SC* human insulin do not have. Note that normalised concentration reflects both free and albumin bound OI338; the majority of OI338 will be associated with albumin.

The simulations were based on data for the insulin analogues tested in dogs and the PK profiles were normalized to the AUC.

For the peak to trough simulations the absorption and volume of distribution was kept constant and clearance was changed dependent of the elimination half-life. In the supplementary table 4 the PK parameters applied for the simulations in Supplementary Fig. 6 is listed.

Parameter	Description	HI	OI338
KV	Distribution volume [L/kg]	0.217	0.11
FP	Plasma fraction of distribution volume	0.635	0.399
Q	Transcompartmental clearance [L/min]	0.0466	0.00203
KP	Plasma compartment elimination rate constant [min^{-1}]	0.142	0.00176
TABS	Absorption time [min] (oral administration only)	30	30
BA	Bioavailability (oral administration only)	0.03	0.03
KS	Depot-1-to-depot-2 transfer rate constant [min^{-1}] (subcutaneous administration only)	0.001	0.001
KF	Depot-2-to-central transfer rate constant [min^{-1}] (subcutaneous administration only)	0.01	0.01
W	Body weight of a dog [kg]	15	15

Supplementary Table 4. Summary of pharmacokinetics parameters used for simulations of *PO* and *SC* administration for human insulin and OI338.

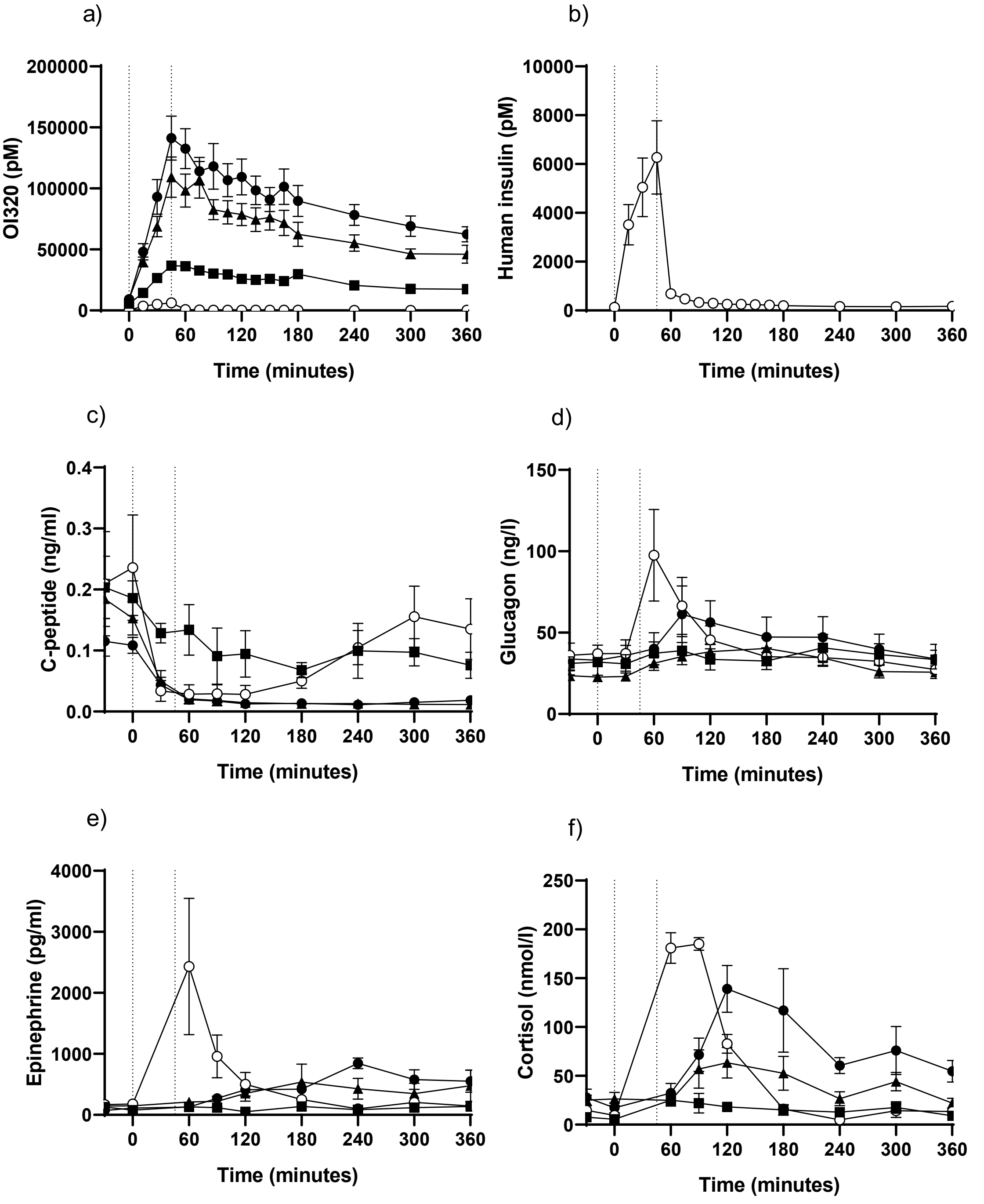

Supplementary Figure 7. Arterial concentrations of OI320 (a) and human insulin (b) during and after the 45 min intraportal insulin infusions on the fourth day. The concentrations of human insulin (b) are also plotted together with the concentrations of OI320 (a). Changes in arterial concentrations of C-peptide (c), glucagon (d), epinephrine (e) in response to the steady state situation. IO320 was infused in three different doses (40 pmol/kg/min (black squares), 80 pmol/kg/min (black triangles) and 120 pmol/kg/min (black circles) (n = 5 for each dose; mean +/- S.E.M.). and human insulin was infused in one dose (24 pmol/kg/min, white circles (n = 3; mean +/- S.E.M.)). The dose of human insulin was set to 60% of the maintenance dose of OI320 (40 pmol/kg/min) in order not to induce too severe hypoglycaemia. The vertical lines in Fig. 7 a-d indicate the portal insulin infusion period from 0-45 min.

Supplementary References

1 Blundell, T. L., Dodson, G. G., Hodgkin, D. C. & Mercola, D. A. Insulin: The structure in the crystal and its reflection in chemistry and biology. *Adv. Protein Chem.* **26,** 279-402 (1972).

2 Schrödinger, LLC. *The PyMOL Molecular Graphics System, Version 1.8* (2015).

3 Fujita-Yamaguchi, Y., Choi, S., Sakamoto, Y. & Itakura, K. Purification of insulin receptor with full binding activity. *J. Biol. Chem.* **258**, 5045-5049 (1983).

4 Slaaby, R. et al. Hybrid receptors formed by insulin receptor (IR) and insulin-like growth factor I receptor (IGF-IR) have low insulin and high IGF-1 affinity irrespective of the IR splice variant. *J. Biol. Chem.* **281**, 25869-25874, doi:10.1074/jbc.M605189200 (2006).

5 Glendorf, T. et al. Systematic evaluation of the metabolic to mitogenic potency ratio for B10-substituted insulin analogues. *PLoS ONE* **7**, doi:10.1371/journal.pone.0029198 (2012).

6 Vienberg, S. G. et al. Receptor-isoform-selective insulin analogues give tissue-preferential effects. *Biochem. J.* **440**, 301-308, doi:10.1042/BJ20110880 (2011).

7 Finney, D. J. Radioligand assay. *Biometrics* **32**, 721-740, doi:10.2307/2529258 (1976).

8 Vølund, A. Application of the four-parameter logistic model to bioassay: Comparison with slope ratio and parallel line models. *Biometrics* **34**, 357-365, doi:10.2307/2530598 (1978).

9 Seglen, P. O. Preparation of isolated rat liver cells. *Methods Cell Biol.* **13**, 29-83 (1976).

10 Lowry, O. H. & Passonneau, J. V. in *A Flexible System of Enzymatic Analysis* (Academic Press, New York, 1972). doi:10.1016/B978-0-12-457950-7.X5001-3.

11 Rodbell, M. Metabolism of isolated fat cells. I. Effects of hormones on glucose metabolism and lypolysis. *J. Biol. Chem.* **239**, 375-380 (1964).

12 Moody, A. J., Stan, M. A., Stan, M. & Gliemann, J. A simple free fat cell bioassay for insulin. *Horm. Metab. Res.* **6**, 12-16, doi:10.1055/s-0028-1093895 (1974).

13 Knudsen, L. et al. Agonism and antagonism at the insulin receptor. *PLoS ONE* **7**, doi:10.1371/journal.pone.0051972 (2012).

14 Kurtzhals, P. et al. Albumin binding of insulins acylated with fatty acids: Characterization of the ligand-protein interaction and correlation between binding affinity and timing of the insulin effect in vivo. *Biochem. J.* **312**, 725-731 (1995).
