# Supplementary material for: Molecular engineering of safe and efficacious oral basal insulin
Source: Nat Commun. 2020 Jul 27;11:3746. doi: 10.1038/s41467-020-17487-9 (PMC7385171; doi:10.1038/s41467-020-17487-9)
Supplement: Supplementary file 2 — Reporting Summary [file 41467_2020_17487_MOESM2_ESM.pdf]

natureresearch

Corresponding author(s): Thomas Kjeldsen

Last updated by author(s): 2020/06/26

Reporting Summary

Nature Research wishes to improve the reproducibility of the work that we publish. This form provides structure for consistency and transparency in reporting. For further information on Nature Research policies, seeAuthors & Referees and theEditorial Policy Checklist .

Statistics

For all statistical analyses, confirm that the following items are present in the figure legend, table legend, main text, or Methods section.

n/a	Confirmed	
☐	☒	The exact sample size (*n*) for each experimental group/condition, given as a discrete number and unit of measurement
☐	☒	A statement on whether measurements were taken from distinct samples or whether the same sample was measured repeatedly
☒	☐	The statistical test(s) used AND whether they are one- or two-sided<br>*Only common tests should be described solely by name; describe more complex techniques in the Methods section.*
☒	☐	A description of all covariates tested
☒	☐	A description of any assumptions or corrections, such as tests of normality and adjustment for multiple comparisons
☐	☒	A full description of the statistical parameters including central tendency (e.g. means) or other basic estimates (e.g. regression coefficient) AND variation (e.g. standard deviation) or associated estimates of uncertainty (e.g. confidence intervals)
☒	☐	For null hypothesis testing, the test statistic (e.g. *F*, *t*, *r*) with confidence intervals, effect sizes, degrees of freedom and *P* value noted<br>*Give P values as exact values whenever suitable.*
☒	☐	For Bayesian analysis, information on the choice of priors and Markov chain Monte Carlo settings
☒	☐	For hierarchical and complex designs, identification of the appropriate level for tests and full reporting of outcomes
☒	☐	Estimates of effect sizes (e.g. Cohen's *d*, Pearson's *r*), indicating how they were calculated

Our web collection on statistics for biologists contains articles on many of the points above.

Software and code

Policy information about availability of computer code

Data collection	HKL2000 v715; XDS VERSION January 10, 2014
Data analysis	Phoenix (1.15) including AutoSol and Phaser; Coot (0.8.9.2); Graph Pad Prism (Version 8.0.2), Phoenix® WinNonlin® version 8.1

For manuscripts utilizing custom algorithms or software that are central to the research but not yet described in published literature, software must be made available to editors/reviewers. We strongly encourage code deposition in a community repository (e.g. GitHub). See the Nature Research guidelines for submitting code & software for further information.

Data

Policy information about availability of data

All manuscripts must include a data availability statement. This statement should provide the following information, where applicable:

- Accession codes, unique identifiers, or web links for publicly available datasets
- A list of figures that have associated raw data
- A description of any restrictions on data availability

Crystal structure coordinates and structure factors were deposited in the Protein Data Bank (PDB) under the following accession codes: 6S4I (OI338) and 6S4J (OI320). Furthermore, human insulin (Protein Data Bank ID 6S34) was used for comparison. The authors declare that the data supporting the findings of this study are available within the paper and its supplementary information files. Raw data are available from the corresponding author upon request

Field-specific reporting

Please select the one below that is the best fit for your research. If you are not sure, read the appropriate sections before making your selection.

Life sciences study design

All studies must disclose on these points even when the disclosure is negative.

Sample size	Sample size in these preclinical studies was chosen as compromise between sufficient number of replicates (n=8 dogs per group) and justification of number of animal used (it was not considered justifiable to use larger group of dogs for preclinical studies, where possible several studies were combined to increase the sample number).
Data exclusions	No data points were excluded
Replication	Several studies were combined to increase the sample number, for example oral availability in dogs for OI338 (n=40) was calculated by combining results from five independent studies (8 dogs in each study); all studies were successful and reproducible.
Randomization	No randomization was included in our protocols as in our studies we tested for absorption of the drug (not an effect) and all animals were treated identically. Furthermore, we have screened the analogues 'one-at-the-time' and therefore we have not randomized the studies.
Blinding	Although the animal technicians were not blinded, the analysis technicians were blinded to treatment status when analyzing the individual samples from the entire study.

Reporting for specific materials, systems and methods

We require information from authors about some types of materials, experimental systems and methods used in many studies. Here, indicate whether each material, system or method listed is relevant to your study. If you are not sure if a list item applies to your research, read the appropriate section before selecting a response.

Materials & experimental systems

n/a	Involved in the study	
☐	☒	Antibodies
☐	☒	Eukaryotic cell lines
☒	☐	Palaeontology
☐	☒	Animals and other organisms
☒	☐	Human research participants
☒	☐	Clinical data

Methods

n/a	Involved in the study	
☒	☐	ChIP-seq
☒	☐	Flow cytometry
☒	☐	MRI-based neuroimaging

Antibodies

Antibodies used	Two monoclonal antibodies were used in a sandwich LOCI assay; both antibodies were produced internally at Novo Nordisk
Validation	Lower limit of qauntittaion for OI320 and OI338 in dog plasma assay was determined to be 30 pM and 50 pM, respectively. Cross reactivity to canin insulin in this assay was <1%

Eukaryotic cell lines

Policy information about cell lines

Cell line source(s)	CHO cell line overexpresing human insulin receptor was prepared at Novo Nordisk in 1996 from CHO K1 strain
Authentication	Cells were not authenticated
Mycoplasma contamination	Mycoplasma contamination was not tested
Commonly misidentified lines (See ICLAC register)	*Name any commonly misidentified cell lines used in the study and provide a rationale for their use.*

Animals and other organisms

Policy information about studies involving animals; ARRIVE guidelines recommended for reporting animal research

Laboratory animals	Healthy and conscious male Beagle dogs (3-4 years old), healthy and conscious male Mongrel dogs (11-14 months old), Healthy and conscious female domestic pigs (Landrace × Yorkshire × Duroc (LYD), 4-5 months old), healthy and conscious male Sprague Dawley rats (13-15 weeks old)
Wild animals	None

Field-collected samples	None
Ethics oversight	All animal studies, expect those involving mongrel dogs, was approval by the Danish "Animal Experiments Inspectorate" which is part of the "Ministry of Environment and Food Denmark. Studies with mongrel dogs was approved by Vanderbilt University Institutional Animal Care and Use Committee USA, and housing and care followed American Association for Laboratory Animal Care guidelines. All animal study protocol was approved by Novo Nordisk Ethical Review Committee, Denmark

Note that full information on the approval of the study protocol must also be provided in the manuscript.
